# Lifestyle Behaviours, Dietary Habits, and Health Awareness Among Residential Undergraduate Medical Students: A Cross-Sectional Study

**DOI:** 10.7759/cureus.110959

**Published:** 2026-06-16

**Authors:** Souradeep Saha, Anita Teli, Gouher Banu Shaikh, Lata Mullur

**Affiliations:** 1 Physiology, Shri B. M. Patil Medical College, Hospital and Research Centre, BLDE (Deemed to be University), Vijayapura, IND

**Keywords:** diet, lifestyle behaviour, medical students, nutrition awareness, physical activity, sleep

## Abstract

Introduction

Medical students are expected to possess sound knowledge of healthy lifestyle practices; however, demanding academic schedules, irregular routines, and hostel-based living may negatively influence their dietary habits, sleep patterns, physical activity, and overall health behaviours. This can lead to a discrepancy between health awareness and actual practices, making this group particularly relevant for lifestyle-related research.

Aim

This study aimed to evaluate lifestyle behaviours, dietary habits, and health awareness among residential undergraduate medical students.

Objectives

The following are the objectives of this study: to assess dietary habits and breakfast-related behaviours, to evaluate sleep patterns and screen time behaviour, to assess levels of physical activity, to examine awareness regarding food ingredients and nutrition labelling, and to identify the gap between health awareness and actual lifestyle practices.

Methods

A quantitative, cross-sectional descriptive study was conducted at Shri B. M. Patil Medical College, Hospital and Research Centre, BLDE (Deemed to be University), Vijayapura, Karnataka, India. A total of 199 residential undergraduate medical students aged 18-25 years from all academic phases participated. Data were acquired using a structured, self-administered questionnaire developed by the authors and validated using the Content Validity Index (CVI) methodology, assessing dietary habits, sleep patterns and screen time behaviour, physical activity, and awareness of food ingredients and nutrition labels. Data were analysed using descriptive statistics. Categorical variables were summarised as frequency and percentage (n (%)). No inferential statistical tests were performed.

Results

A total of 130 (65.3%) students reported sometimes or often skipping breakfast, while 21 (10.6%) reported always skipping breakfast. A total of 91 (45.7%) students reported sleeping less than six hours per night, and 188 (94.5%) used digital devices before bedtime. Although 139 (69.8%) students reported engaging in physical activity, only 50 (25.1%) exercised daily. Regular reading of nutrition labels was reported by 77 (38.7%) students. Health awareness was higher than consistent healthy practices across all domains.

Conclusion

Residential medical students demonstrated moderate to high health awareness and poor adherence to healthy lifestyle behaviours, indicating an ongoing knowledge-practice gap and the need for institution-based interventions.

## Introduction

Medical students are expected to promote healthy lifestyles; however, the demanding nature of medical education often interferes with the adoption of healthy personal behaviours. Long academic hours, psychological stress, irregular schedules, hostel-based living, and extensive screen time contribute to unhealthy dietary practices, disrupted sleep, reduced physical activity, and inconsistent use of nutrition information [1-4]. Although individual lifestyle components, such as diet or sleep, have been studied in isolation, little attention has been given to the combined assessment of lifestyle behaviours among medical students living in residential campus settings [1,2,5]. Understanding these patterns is important, as health behaviours established during professional training may continue into later clinical practice and influence the credibility of physicians as health role models [6].

Dietary habits, sleep patterns, screen time behaviour, physical activity, and nutrition awareness are important and interconnected components of a healthy lifestyle. Among residential medical students, these behaviours may be influenced by common factors such as hostel living, academic workload, irregular schedules, psychological stress, and increased reliance on digital devices. Therefore, evaluating these domains together may provide a more comprehensive understanding of lifestyle behaviours and identify potential areas for institutional health promotion interventions.

The aim of this study was to evaluate lifestyle behaviours, dietary habits, and health awareness among residential undergraduate medical students. The specific objectives were to assess dietary habits and breakfast-related behaviours, evaluate sleep patterns and screen time behaviour, assess levels of physical activity, examine awareness regarding food ingredients and nutrition labelling, and identify potential gaps between health awareness and actual lifestyle practices. Findings from this study may help guide future institutional wellness strategies for medical students [7].

## Materials and methods

Design

This research is a quantitative, cross-sectional, descriptive study designed to evaluate lifestyle behaviours, dietary habits, and health awareness among medical students. A cross-sectional design was adopted to capture prevailing lifestyle patterns at a single point in time within a defined academic setting.

Setting

The study was conducted at Shri B. M. Patil Medical College, Hospital and Research Centre, BLDE (Deemed to be University), Vijayapura, Karnataka, India. The institution offers on-campus hostel accommodation to students in all phases of the undergraduate medical programme, and this creates a residential academic environment that is suitable for the assessment of lifestyle behaviours which are influenced by medical training and hostel life.

Sample

The study population comprised undergraduate medical students from all academic phases who were residing in the campus hostels during the study period. Data collection was conducted between January 2026 and March 2026. The inclusion criteria were hostel residence and willingness to participate. Interns not residing in hostels and students who did not complete the questionnaire were excluded.

Approximately 500 hostel-residing undergraduate medical students were eligible during the study period. Convenience sampling was employed, and participation was voluntary. A total of 199 students aged 18-25 years completed the survey and were included in the final analysis. Owing to the non-probability sampling approach, the findings should be interpreted as descriptive and exploratory.

Measures

Data were collected using a structured, self-administered questionnaire, developed in English to assess four lifestyle domains: (1) dietary habits and breakfast habits, such as frequency of taking breakfast, reasons for skipping meals, preference for hostel or outside food, and frequency of taking outside food; (2) sleep patterns and screen time behaviours, such as average sleep time, bedtime, challenges in sleeping onset or maintenance, electronic device use before bedtime, and time spent on academic and recreational screen time; (3) physical activity, such as type, frequency, and regularity of exercise, perceived impact of medical education on exercise awareness, and interest in additional physical activity-related educational resources; and (4) awareness of food ingredients and nutrition labels, such as frequency of packaged food consumption, label-reading behaviour, perceived knowledge of food ingredients, and the influence of medical education on nutrition awareness.

Most items were categorical or multiple-choice to allow for uniform responses and quantitative analysis. Operational definitions were used in a consistent manner. Breakfast skipping was defined as skipping breakfast at least three times per week. Adequate sleep was defined as a self-reported sleep duration of at least seven hours per night, based on established sleep duration recommendations [8]. Sleep continuity was not specifically assessed. Physical activity was defined as any form of exercise for at least 30 minutes per session, on three or more days per week, in accordance with recommendations from the World Health Organization [9]. Label awareness was classified as often or always reading nutrition labels on packaged foods.

Instrument validation and quality control

The questionnaire was initially developed by the authors following a review of the relevant literature and was subsequently evaluated for content validity by two subject experts from the Department of Community Medicine using the Content Validity Index (CVI) methodology [10]. Expert feedback was used to refine item clarity, relevance, and content coverage before pilot testing. The revised questionnaire was pilot-tested among 10 non-participating undergraduate medical students to assess comprehensibility and logical flow. Based on participant feedback, minor revisions were made to simplify question wording, improve clarity, and remove potentially ambiguous items. Following these refinements, the final questionnaire consisting of 43 items was administered to the study participants. The complete final questionnaire is provided in the Appendices.

The content validity assessment demonstrated satisfactory validity, with a Scale-Level Content Validity Index based on average agreement (S-CVI/Ave) of 0.988 and a Scale-Level Content Validity Index based on universal agreement (S-CVI/UA) of 0.953. Data quality was maintained through careful data entry and random cross-checking of approximately 10% of records. Missing data were minimal and did not materially affect the analysis.

Data collection procedure

Data collection was conducted inside the hostels and during break time after the academic sessions to avoid disruption. Students completed the questionnaire on their own without supervision from faculty to minimise response bias. Participation was voluntary, no personal identifiers were collected, and completed questionnaires were checked on-site for completeness.

Analysis

Data were entered in Microsoft Excel (Microsoft Corporation, Redmond, Washington, United States) and analysed with the help of descriptive statistics. Categorical variables were summarised as frequencies and percentages. No inferential statistical tests were conducted because the nature of the study was exploratory and descriptive. Analysis was based on identifying common behavioural patterns and gaps between health awareness and actual health practices.

Ethical considerations

Ethical approval was obtained before the initiation of the study from the Institutional Ethics Committee of BLDE (Deemed to be University) with approval number BLDE(DU)/IEC-SBMPMC/390/2025-26 on 09/01/2026. Informed consent was obtained from all participants. Confidentiality and anonymity were ensured throughout the study, and participants were informed that they could withdraw from the study at any time without penalty.

## Results

Data from 199 residential undergraduate medical students across all academic phases were analysed. Demographic data is presented below in Table 1.

**Table 1:** Demographic characteristics of the study participants (N=199) Data are presented as n (%).

Variable	Category	n (%)
Gender	Male	105 (52.8)
	Female	94 (47.2)
Academic year	First year	60 (30.2)
	Second year	52 (26.1)
	Third year	54 (27.1)
	Final year	33 (16.6)

A total of 199 hostel-residing undergraduate medical students participated in the study. Of these, 105 (52.8%) were males, and 94 (47.2%) were females. Participants were distributed across academic years as follows: first year, 60 (30.2%); second year, 52 (26.1%); third year, 54 (27.1%); and final year, 33 (16.6%).

All questionnaires were completed and included in the final analysis. The results from the four lifestyle domains, namely, dietary habits, sleep and screen time behaviour, physical activity, and awareness of nutrition labels, are summarised in the figures below.

Breakfast and dietary habits

**Figure 1:**
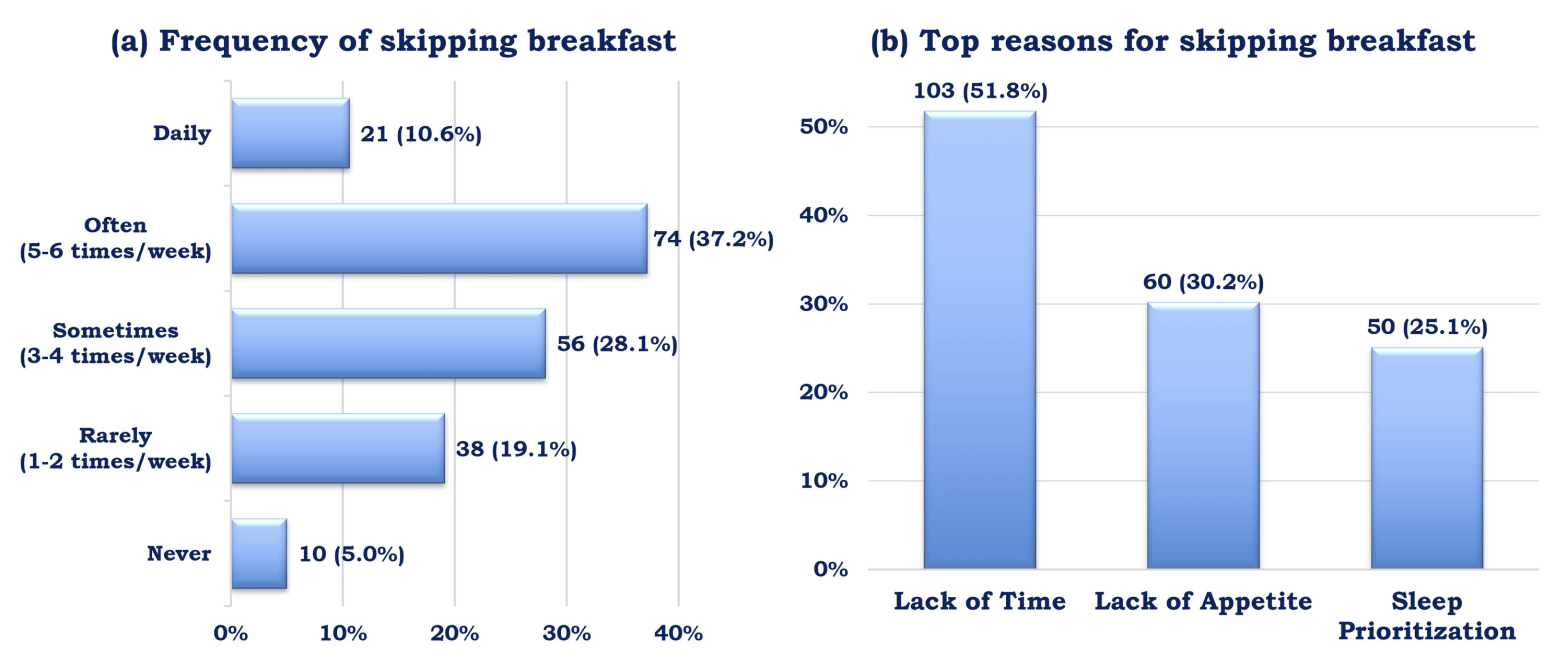
Breakfast skipping behaviour among undergraduate medical students. (a) Frequency of breakfast skipping among study participants. (b) Common reasons for skipping breakfast among undergraduate medical students Data are presented as frequency (n (%)) among 199 participants. Multiple responses were permitted for reasons for skipping breakfast.

As indicated in Figure 1, breakfast skipping was a common practice among the participants. A total of 130 (65.3%) students reported sometimes or often skipping breakfast, while 21 (10.6%) reported always skipping breakfast. The most common reasons given for not eating breakfast were lack of time, 103 (51.8%), lack of appetite, 60 (30.2%), and preference for more sleep, 50 (25.1%). Only the three most frequently reported reasons are displayed in the figure for clarity. A smaller proportion, 30 (15.1%), reported dieting or weight-control intention as a reason for skipping breakfast.

Traditional breakfast items such as idli, dosa, vada, and poha were consumed by 135 (67.8%) students, while bread or toast, eggs, tea or coffee, and fruits were each consumed by 100 (50.3%) students. With regard to food sources, 120 (60.3%) students reported occasional or regular avoidance of hostel mess food in favour of outside food. The major reasons given were dissatisfaction with the taste and quality, 139 (69.8%), lack of variety, 119 (59.8%), and preference to eat food from outside, 100 (50.3%). A total of 90 (45.2%) students ate outside food 2-3 times weekly, with fast food, 129 (64.8%), ethnic foods, 109 (54.8%), and street food, 80 (40.2%), being the most popular food categories. A total of 60 (30.2%) students said they actively chose healthier food options.

Sleep patterns and screen time behaviour

**Figure 2:**
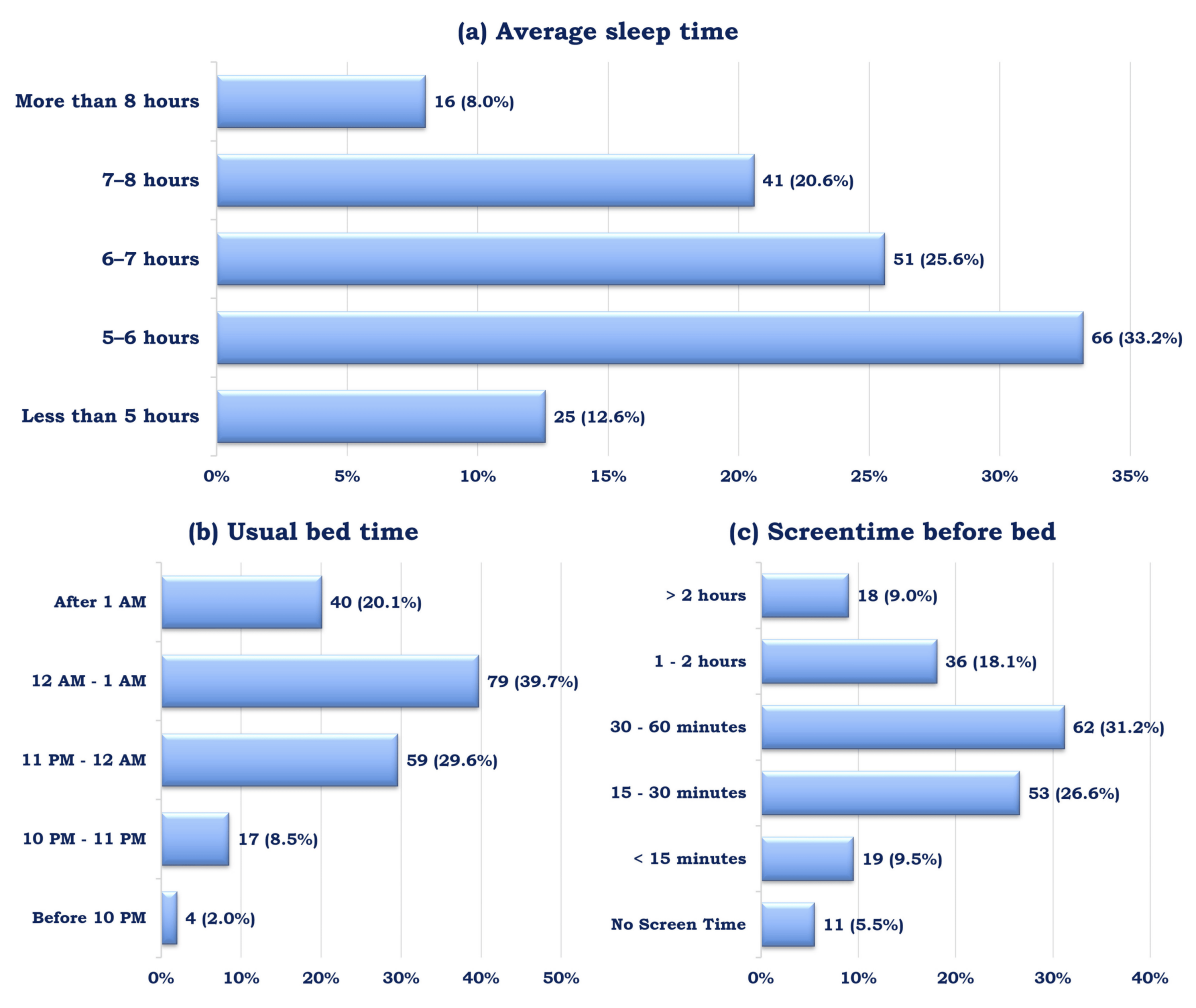
Sleep patterns and screen time behaviour among undergraduate medical students. (a) Average sleep duration on a typical weekday. (b) Usual bedtime on a typical weekday. (c) Duration of screen use immediately before bedtime Data are presented as frequency (n (%)) among 199 participants.

The sleep-related behaviours are described in Figure 2. A total of 91 (45.7%) students reported sleeping less than six hours per night, including 25 (12.6%) who reported sleeping less than five hours. Only 57 (28.6%) participants met the recommended sleep duration of seven hours or more. Bedtime was delayed for a majority of students, with 79 (39.7%) reporting sleep onset time between midnight and 1:00 a.m. and 40 (20.1%) reporting sleep onset time after 1:00 a.m.

Sleep disturbances were common, with 129 (64.8%) students experiencing difficulty falling asleep or maintaining sleep. Pre-sleep screen use was reported by 188 (94.5%) students using a mobile phone or laptop before bedtime. A total of 116 (58.3%) students reported using screens for at least 30 minutes before sleep, and 54 (27.1%) reported more than one hour of screen time before bedtime.

Academic screen exposure was significant, with 60 (30.2%) students reporting a daily screen exposure of 2-3 hours and 50 (25.1%) reporting more than three hours per day on digital learning platforms. Recreational screen use was also high, with 80 (40.2%) students using social media and entertainment platforms for one to two hours per day and 60 (30.2%) using them for two to three hours a day.

Physical activity

**Figure 3:**
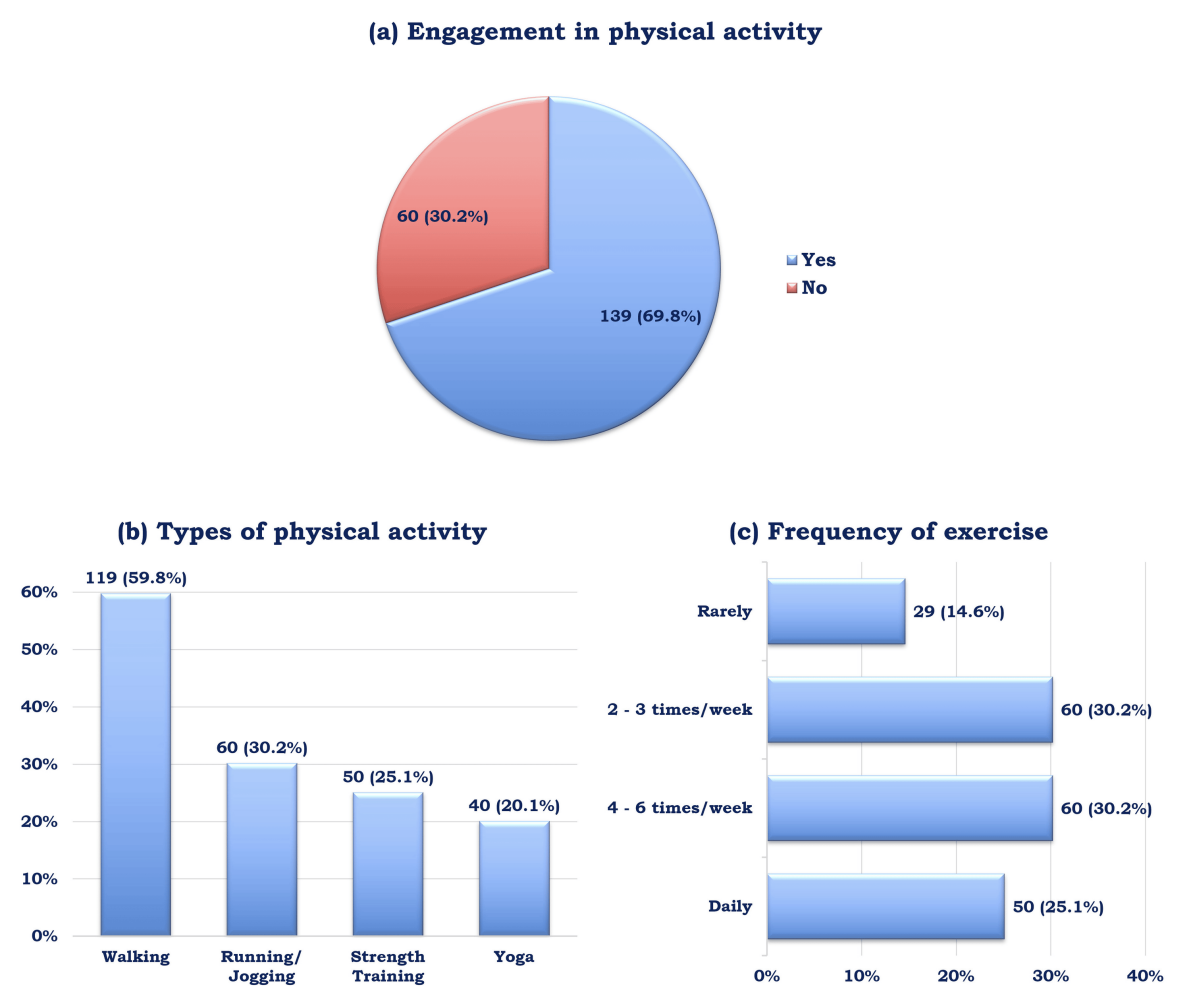
Physical activity patterns among undergraduate medical students. (a) Participation in regular physical activity. (b) Types of physical activities performed by students. (c) Frequency of exercise among students engaging in physical activity Data are presented as frequency (n (%)) among 199 participants. Multiple responses were permitted for the type of physical activity.

Physical activity patterns are shown in Figure 3. A total of 139 (69.8%) students reported engaging in physical activity of any kind. Walking was the most common activity, 119 (59.8%), followed by running or jogging, 60 (30.2%), strength training, 50 (25.1%), and yoga, 40 (20.1%). Only 50 (25.1%) students reported exercising daily, while 120 (60.3%) exercised two to six times a week.

A total of 159 (79.9%) students reported increased awareness of physical fitness due to medical education; however, only 50 (25.1%) reported daily exercise. Interest in additional educational resources pertaining to exercises was indicated by 129 (64.8%) students.

Food ingredient and nutrition label awareness

**Figure 4:**
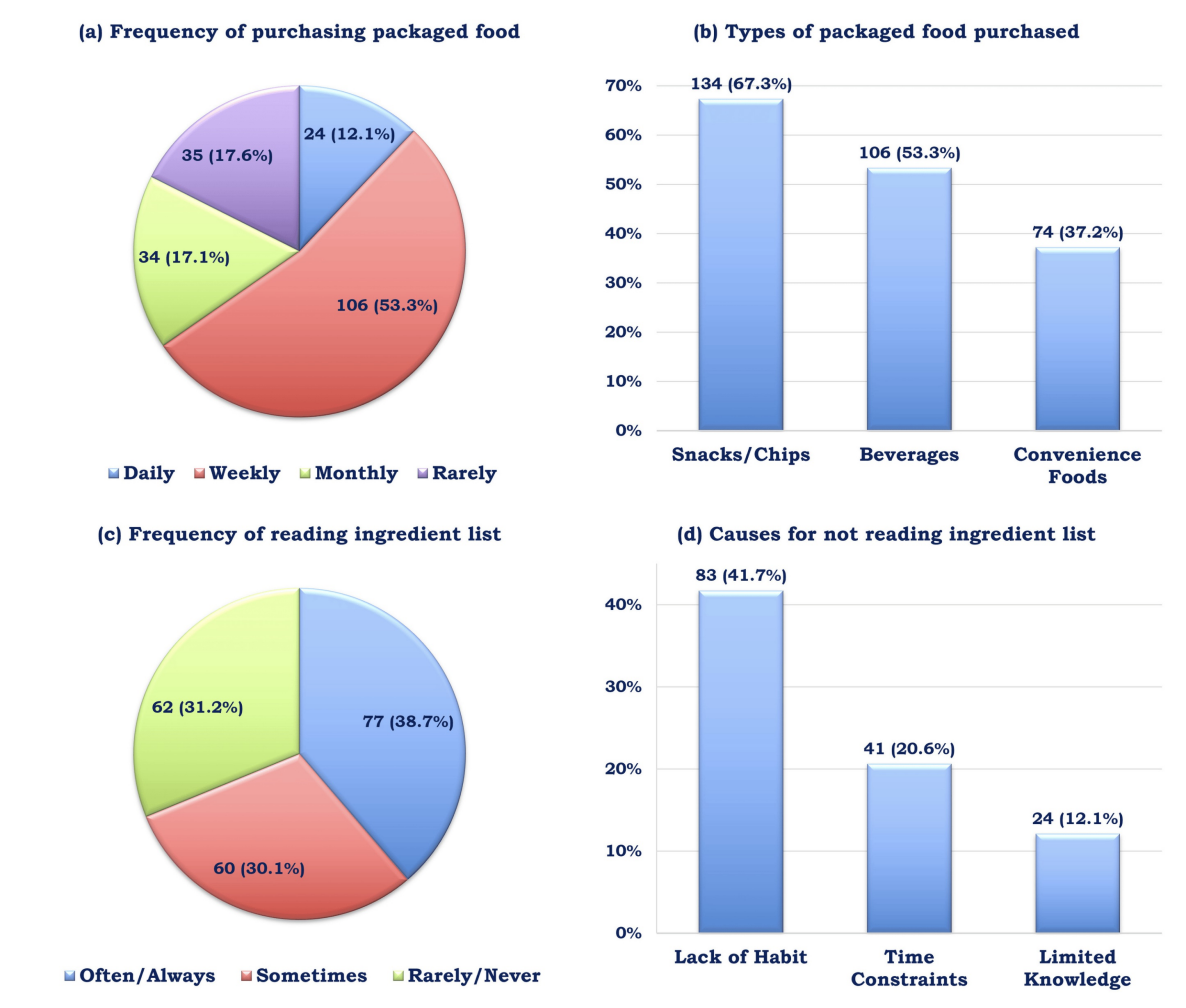
Awareness and utilisation of food ingredient information among undergraduate medical students. (a) Frequency of purchasing packaged food. (b) Common types of packaged food purchased by students. (c) Frequency of reading ingredient labels on packaged food products. (d) Common reasons for not reading ingredient labels Data are presented as frequency (n (%)) among 199 participants. Multiple responses were permitted for the type of packaged food purchased and reasons for not reading ingredient labels.

Patterns related to nutritional awareness are presented in Figure 4. Packaged food consumption was common, with 106 (53.3%) students buying such products on a weekly basis and 34 (17.1%) on a monthly basis. Commonly consumed packaged foods were snacks or chips, 134 (67.3%), beverages, 106 (53.3%), and convenience foods, 74 (37.2%).

Regular reading of nutrition labels was reported by 77 (38.7%) students, 60 (30.2%) read nutrition labels occasionally, and 62 (31.1%) rarely or never read nutrition labels. The most common reasons reported for not reading labels were lack of habit, 83 (41.7%), limited time, 41 (20.6%), and difficulty in understanding the information, 24 (12.1%). A total of 119 (59.8%) students assessed their level of knowledge of food ingredients as moderate to high. A total of 149 (74.9%) students reported increased awareness due to medical education, while consistent label-reading behaviour was reported by 77 (38.7%) students.

## Discussion

This study provides an overview of lifestyle behaviours, dietary habits, sleep and screen time patterns, physical activity, and awareness of nutrition among residential undergraduate medical students of a tertiary South Indian medical college. Despite moderate to high levels of awareness in several health domains, there is consistent poor adherence to healthy lifestyle practices, suggesting a potential gap between health awareness and healthy lifestyle practices.

Dietary habits and breakfast skipping

Breakfast skipping was reported by a substantial proportion of participants, and this finding is consistent with national and international research among medical and university students [3,5]. Time constraints, desire for more sleep, and lack of appetite were the most frequent reasons for not eating breakfast, which is similar to the findings that have been reported as determinants of breakfast skipping among Asian and European student populations [1,3]. Hostel living and strict academic schedules are also factors that lead to non-regular meal patterns and reinforce convenience-driven food choices over nutritional considerations [5].

The preference for food obtained outside the hostel mess, particularly fast food and street food options, has also been reported in previous studies in India [5,11]. In the present study, outside food encompassed a broad range of food choices, including fast food, street food, ethnic cuisine, and healthier alternatives. Therefore, the nutritional quality of all outside food cannot be assumed to be uniform. Nevertheless, frequent consumption of certain energy-dense and nutrient-poor food options available outside the hostel environment may contribute to unhealthy dietary patterns and long-term metabolic risk. Although many students demonstrated awareness of healthier food choices, consistent adoption of these practices was not uniformly observed, highlighting the complexity of behavioural change in demanding academic environments.

Sleep patterns and screen time behaviour

Sleep deprivation was highly prevalent, and a large percentage of students reported less than the recommended amount of sleep [8]. Similar patterns have been documented for the global population of medical students and often attributed to academic workload, studying late at night, and the use of digital devices [2,12]. The delayed bedtime in this study, along with extensive pre-sleep screen exposure, is consistent with evidence from the digital health literature, suggesting potential disruption of circadian rhythms [4,13].

The near-universal use of electronic devices before bedtime and prolonged screen exposure for academic and recreational purposes may be associated with difficulty in sleep initiation and maintenance due to blue light exposure [4]. Inadequate exposure to sunlight may also contribute to sleep deprivation by impairing circadian entrainment [2]. Previous research has demonstrated the association between excessive screen time, poor sleep quality, cognitive impairment, and emotional distress in young adults [14,15]. These findings support the importance of targeted sleep hygiene education, promotion of adequate sunlight exposure, and digital well-being interventions within the medical campus environment.

Physical activity

Although the majority of students reported being engaged in some form of physical activity, regular and consistent activity was lacking. Such a gap between awareness and sustained physical activity practice has been reported widely among medical trainees and young adults globally [16-18]. Time constraints, academic pressure, and limited access to structured exercise facilities have been commonly identified as barriers to regular physical activity in university and medical college settings [17].

The finding that medical education raised awareness of physical fitness but did not lead to proportional behavioural change only underlines the limitation of knowledge-based approaches. Previous evidence suggests that institutional support, structured programmes, and environmental modifications are necessary to promote sustained physical activity among students [18]. The expressed interest in fitness workshops, mobile applications, and educational resources observed in this study further indicates receptiveness to such interventions.

Nutrition awareness and label-reading behaviour

Awareness of food ingredients and nutrition labels was moderate among participants; however, consistent label-reading behaviour was low. This observation is consistent with the existing literature which suggests that nutrition knowledge does not always translate into healthier purchasing or consumption behaviours [19]. Lack of habit, time constraints, and difficulty in interpreting label information were the most commonly reported barriers, which were similar to findings in other student populations [19].

Medical education seemed to positively influence nutrition awareness; however, this awareness was inconsistently applied in everyday food choices. Prior studies suggest that incorporating practical and skills-based nutrition education is more effective than theoretical instruction alone in improving dietary decision-making [19]. Incorporating applied nutrition literacy early in medical training may, therefore, help bridge the gap between knowledge and practice.

Integrated lifestyle patterns and implications

Across all four lifestyle domains, a recurring pattern of relatively high levels of health awareness but poor levels of adherence to healthy behaviours was observed. The concurrent presence of unhealthy behaviours, including late-night screen use, inadequate sleep, breakfast skipping, and irregular physical activity, was observed among participants. These behaviours may coexist within the same population; however, the present study did not formally assess behavioural clustering. Similar patterns involving multiple lifestyle risk behaviours have been reported among medical and university students in previous studies [14,15].

This pattern is of particular concern in light of evidence that physicians following healthy lifestyles are more likely to provide appropriate counselling to patients regarding preventive health behaviours [6]. As future healthcare providers, the personal health practices of medical students may therefore influence their professional credibility and patient outcomes. Therefore, promotion of healthy lifestyles among medical students is not only an individual concern but a professional and institutional responsibility as well.

Strengths and limitations

The strengths of this study include the comprehensive assessment of multiple lifestyle domains among hostel-residing undergraduate medical students using a validated questionnaire. However, several limitations should be acknowledged. The cross-sectional design precludes causal inference, and the use of self-reported data introduces the potential for recall bias and social desirability bias. In addition, convenience sampling and voluntary participation may have introduced selection bias, as students with greater interest in health and lifestyle issues may have been more likely to participate. Furthermore, the single-institution setting limits the generalisability of the findings to non-residential medical students, interns, and students from other medical colleges. Therefore, the results should be interpreted as descriptive and exploratory.

The study employed descriptive statistical methods and did not perform analyses to assess associations, co-occurrence patterns, or clustering among lifestyle behaviours. Therefore, the findings should not be interpreted as evidence of behavioural clustering. Additionally, anthropometric measurements, body mass index, stress levels, mental health status, caffeine intake, academic workload, and objective measures of physical activity and sleep were not assessed and may have influenced the observed lifestyle behaviours. Nevertheless, the internal validity of the study is strengthened by the relatively homogeneous residential and academic environment of the participants. Future studies may benefit from examining associations between lifestyle behaviours and demographic or academic characteristics using inferential statistical methods.

## Conclusions

This study showed that the residential undergraduate medical students had moderate to high awareness of healthy lifestyle practices, but poor adherence in key areas of diet, sleep, physical activity, and nutrition label use. The study observed the concurrent presence of breakfast skipping, insufficient sleep, high screen exposure, and inconsistent physical activity among residential medical students despite relatively high levels of health awareness. These findings highlight a potential knowledge-practice gap within the residential medical training environment.

## Appendices

**Table 2:** Final validated questionnaire used for the assessment of lifestyle behaviours, dietary habits, sleep patterns, physical activity, and health awareness among residential undergraduate medical students The questionnaire was developed by the authors based on a review of relevant literature and subsequently validated using the Content Validity Index (CVI) methodology. The final validated questionnaire consisted of 43 items and was administered to all study participants. Questions covered dietary habits, sleep patterns and screen time behaviour, physical activity, and awareness regarding food ingredients and nutrition labelling.

A Survey on Lifestyle and Health Behaviour Patterns Among Residential Undergraduate Students
SECTION 1: BREAKFAST HABITS
1. How often do you skip breakfast?
□ Never - I always eat breakfast
□ Rarely - I skip breakfast only occasionally
□ Sometimes - I miss breakfast a few times a week
□ Often - I skip breakfast regularly
□ Always - I never eat breakfast
2. What is the main reason for skipping breakfast? (Applicable only if skipped) (Select all that apply)
□ Lack of Time - I'm too busy in the morning to have breakfast
□ Lack of Appetite - I don't feel hungry in the morning
□ Dieting or Weight Control - I skip breakfast as part of a weight loss or dietary plan
□ Prefer to Sleep Longer - I prioritize sleeping over having breakfast
□ Food Preferences - I don't like the options available for breakfast
□ Health Issues - I have health-related reasons or medical conditions affecting my appetite
□ Other (please specify): __________
3. If breakfast is not skipped, what do you typically prefer to have for breakfast? (Select all that apply)
□ Eggs
□ Toast or bread
□ Fruits
□ Yogurt
□ Coffee or tea
□ Idli
□ Dosa
□ Vada
□ Upma
□ Poha
□ Other (please specify): __________
SECTION 2: SLEEP HABITS
4. On average, how many hours of sleep do you get on a typical weekday?
□ Less than 5 hours
□ 5-6 hours
□ 6-7 hours
□ 7-8 hours
□ 8-9 hours
□ More than 9 hours
5. What time do you usually go to bed on a typical weekday?
□ Before 10 PM
□ 10 PM-11 PM
□ 11 PM-12 AM
□ 12 AM-1 AM
□ After 1 AM
6. What time do you usually wake up on a typical weekday?
□ Before 5 AM
□ 5 AM-6 AM
□ 6 AM-7 AM
□ 7 AM-8 AM
□ After 8 AM
7. Do you experience difficulty falling asleep or staying asleep?
□ Always - I have difficulty falling asleep or staying asleep every night
□ Often - I frequently have trouble falling asleep or staying asleep
□ Occasionally - I sometimes experience difficulty with falling asleep or staying asleep
□ Rarely - I rarely have issues with falling asleep or staying asleep
□ Never - I never have difficulty falling asleep or staying asleep
8. Do you use your mobile phone, laptop, or tablet immediately before bedtime?
□ Yes
□ No
9. If yes, how much time do you typically spend using these devices before going to sleep?
□ Less than 15 minutes
□ 15-30 minutes
□ 30-60 minutes
□ 1-2 hours
□ More than 2 hours
□ I do not use devices before going to sleep
10. Do you fall asleep while using your mobile phone, laptop, or tablet?
□ Always - I often fall asleep while using these devices
□ Often - I frequently fall asleep while using these devices
□ Occasionally - I sometimes fall asleep while using these devices
□ Rarely - I rarely fall asleep while using these devices
□ Never - I never fall asleep while using these devices
SECTION 3: SCREEN TIME
11. On average, how many hours per day do you spend on your mobile, laptop, or tablet for academic purposes?
□ Less than 30 minutes
□ 30-60 minutes
□ 1-2 hours
□ 2-3 hours
□ More than 3 hours
12. List three medical educational apps that you use regularly.
□
13. List three medical fitness apps that you use regularly.
□
14. On average, how many hours per day do you spend on your mobile, laptop, or tablet for recreational purposes?
□ Less than 30 minutes
□ 30-60 minutes
□ 1-2 hours
□ 2-3 hours
□ More than 3 hours
15. List the top 3 social media apps that you use regularly? (Select maximum 3 options)
□ Instagram
□ WhatsApp
□ Facebook
□ X (Twitter)
□ Snapchat
□ Telegram
□ Pinterest
□ Any Other: __________
16. List the top 3 video streaming apps that you use regularly? (Select maximum 3 options)
□ YouTube
□ Hotstar
□ Netflix
□ Amazon Prime Video
□ SonyLIV
□ Disney+ Hotstar
□ Any Other: __________
SECTION 4: PERSONAL HYGIENE
17. Do you wash your hand every time before you have food?
□ Yes
□ No
18. What situations prompt you to wash your hands? (Select all that apply)
□ After using the restroom
□ Before eating
□ After coughing or sneezing
□ After being in public places
□ Other (please specify): __________
19. Do you use soap/liquid soap while washing your hands?
□ Always
□ Sometimes
□ Never
20. Are you aware of the recommended duration for handwashing?
□ Yes
□ No
21. What do you think is the most effective way to wash hands?
□ Using soap and water
□ Using hand sanitizer
□ Just rinsing with water
□ Other (please specify): __________
SECTION 5: HOSTEL MESS FOOD VS. OUTSIDE FOOD
22. How often do you skip hostel mess food and consume outside food?
□ Never - I always eat at the hostel mess
□ Rarely - I skip the hostel mess food only on special occasions
□ Occasionally - I eat outside food once in a while, but I still go to the hostel mess regularly
□ Frequently - I often choose outside food over the hostel mess
□ Always - I prefer eating outside food and skip the hostel mess completely
23. What are the main reasons for skipping hostel mess food and opting for outside food? (Select all that apply)
□ Quality of Food - The quality or taste of the hostel mess food is not satisfactory
□ Variety - There is a lack of variety in the hostel mess menu
□ Health Concerns - I have specific dietary or health needs that are not met by the hostel mess food
□ Preference - I prefer the taste or style of outside food
□ Convenience – It's more convenient to get food from outside
□ Social Reasons - I prefer eating with friends outside rather than in the hostel mess
□ Availability - The hostel mess food is not available at times that suit me
□ Cleanliness - Concerns about the hygiene or cleanliness of the hostel mess
□ Other (please specify): __________
24. How do you like to consume outside food?
□ By online food delivery app
□ By physically going to a restaurant
25. How frequently do you consume outside food?
□ 0-1 day per week
□ 2-3 days per week
□ 4-5 days per week
□ 6-7 days per week
26. Do you have specific types of outside food that you prefer? (Select all that apply)
□ Fast Food - Burgers, fries, pizza, etc.
□ Street Food - Tacos, kebabs, chaat, etc.
□ Ethnic Cuisine - Indian, Chinese, Italian, Thai, etc.
□ Healthy Options - Salads, wraps, smoothies, etc.
□ Desserts - Ice cream, pastries, cakes, etc.
□ Snacks - Chips, nuts, sandwiches, etc.
□ Beverages - Coffee, tea, juices, etc.
□ Vegetarian - Plant-based or vegetarian dishes
□ Non-vegetarian - Meat, poultry, seafood dishes
□ Specialty or Gourmet Foods - High-end or unique culinary experiences
□ Other (please specify): __________
SECTION 6: PHYSICAL ACTIVITY
27. What type of physical activities do you do regularly? (Select all that apply)
□ No regular physical activities
□ Walking
□ Running/jogging
□ Cycling
□ Swimming
□ Yoga
□ Strength training (weightlifting, resistance bands)
□ Cardio exercises (e.g., aerobics, HIIT)
□ Outdoor sports (e.g., basketball, soccer)
□ Dancing
□ Home workout routines
□ Other (please specify): __________
28. How frequently do you engage in these physical activities?
□ Daily
□ 4-6 times per week
□ 2-3 times per week
□ Once a week
□ 2-3 times per month
□ Once a month or less
□ Rarely/never
29. Has your medical training influenced your approach to understanding the benefits of exercise?
□ Yes
□ No
30. Do you feel your understanding of exercise and its health benefits has improved during your medical education?
□ Yes
□ No
31. Would you be interested in receiving more education on the health impacts of exercise?
□ Yes
□ No
32. What type of resources would you find most helpful for improving your knowledge about the benefits of exercise? (Select all that apply)
□ Workshops or Seminars - In-person or online events with experts
□ Online Courses - Structured educational content available online
□ Educational Booklets - Printed or digital materials with information on exercise benefits
□ Interactive Tools or Apps - Digital tools or apps that provide exercise information
□ Social Media/Influencers
□ Other (please specify): __________
SECTION 7: FOOD INGREDIENT AWARENESS
33. How often do you purchase packaged food?
□ Daily (e.g., buying lunch or snacks every day)
□ Weekly (e.g., buying packaged food several times a week)
□ Monthly (e.g., buying packaged food once a month)
□ Rarely (e.g., only on special occasions or infrequently)
34. Which of the following types of food items do you purchase regularly? (Select all that apply)
□ Snacks and Chips (Potato Chips, Namkeen, Bhujia, Peanuts, and Roasted Snacks)
□ Beverages (Soft Drinks, Juices, Energy Drinks, Tea, Coffee)
□ Convenience Foods (Instant Noodles, Ready-to-Eat Meals, Microwaveable Rice and Grains)
□ Breakfast Foods (Breakfast Cereals, Instant Oatmeal, Pancake Mix, Yogurt)
□ Condiments and Sauces (Ketchup, Mayonnaise, Chutneys, Pasta Sauces)
□ Baked Goods (Cookies, Bread, Cakes, and Pastries)
□ Dairy Products (Cheese, Milk, Butter, and Margarine)
□ Frozen Foods (Ice Cream, Frozen Vegetables, Frozen Fruits)
□ Canned Goods (Canned Vegetables, Canned Fruits, Canned Beans, Canned Tuna)
□ Health and Special Diet Foods (Gluten-Free Products, Plant-Based Milks, Protein Bars, Low-Sugar or Sugar-Free Products)
35. When purchasing packaged food, how often do you read the ingredient list?
□ Always (e.g., I read the ingredient list every time I buy packaged food)
□ Often (e.g., I read the ingredient list most of the time)
□ Sometimes (e.g., I read the ingredient list occasionally)
□ Rarely (e.g., I rarely read the ingredient list)
□ Never (e.g., I never read the ingredient list)
36. What factors influence your decision to check or not check the ingredient list? (Select all that apply)
□ Time Constraints - I don't have enough time to read the ingredient list
□ Habit - I usually don't think to read the ingredient list
□ Health Considerations - I read the ingredient list to make healthier choices
□ Lack of Knowledge - I don't understand what the ingredients mean
□ Product Branding - I rely on the brand's reputation rather than the ingredient list
□ Recommendations from Others - I follow advice from friends, family, or dietitians
□ Other (please specify): __________
37. How knowledgeable do you feel about the health effects of common food ingredients?
□ Very Knowledgeable - I have a thorough understanding of how various ingredients affect health
□ Somewhat Knowledgeable - I have a general understanding of ingredient effects but not in-depth knowledge
□ Neutral - I have an average understanding but could use more information
□ Slightly Knowledgeable - I know a little about ingredient effects but not enough to make informed choices
□ Not Knowledgeable at All - I have little to no knowledge about the health effects of food ingredients
38. Which of the following ingredients are you aware of and can identify as generally good for health? (Select all that apply)
□ Trans Fats - Found in partially hydrogenated oils
□ Whole Grains - Such as oats, barley, or quinoa
□ High-Fructose Corn Syrup - A type of added sugar
□ Omega-3 Fatty Acids - Found in fish oil or flaxseed oil
□ High-Sodium Foods
□ Vitamins - Such as vitamin C (ascorbic acid) or vitamin D
□ Artificial Preservatives - Such as BHA/BHT or sodium nitrate
□ Natural Sweeteners - Such as honey or maple syrup
□ Artificial Sweeteners - Such as aspartame or saccharin
□ Fiber - Found in vegetables, legumes, and fruits
39. Which of the following ingredients are you aware of and can identify as generally bad for health? (Select all that apply)
□ Trans Fats - Found in partially hydrogenated oils
□ Whole Grains - Such as oats, barley, or quinoa
□ High-Fructose Corn Syrup - A type of added sugar
□ Omega-3 Fatty Acids - Found in fish oil or flaxseed oil
□ High-Sodium Foods
□ Vitamins - Such as vitamin C (ascorbic acid) or vitamin D
□ Artificial Preservatives - Such as BHA/BHT or sodium nitrate
□ Natural Sweeteners - Such as honey or maple syrup
□ Artificial Sweeteners - Such as aspartame or saccharin
□ Fiber- Found in vegetables, legumes, and fruits
40. Has your medical training influenced your approach to reading ingredient lists?
□ Yes
□ No
41. Do you feel your understanding of nutrition and ingredient lists has improved during your medical education?
□ Yes
□ No
42. Would you be interested in receiving more education on interpreting ingredient lists and understanding their health impacts?
□ Yes
□ No
43. What type of resources would you find most helpful for improving your knowledge about food ingredients? (Select all that apply)
□ Workshops or Seminars - In-person or online events with experts
□ Online Courses - Structured educational content available online
□ Educational Handouts or Booklets - Printed or digital materials with information on ingredients
□ Interactive Tools or Apps - Digital tools or apps that provide ingredient information
□ Social Media/Influencers
□ Other (please specify): __________
